# Long-term treatment of clarithromycin at a low concentration improves hydrogen peroxide-induced oxidant/antioxidant imbalance in human small airway epithelial cells by increasing Nrf2 mRNA expression

**DOI:** 10.1186/s40360-017-0119-8

**Published:** 2017-02-25

**Authors:** Kuninori Iwayama, Ayuko Kusakabe, Keisuke Ohtsu, Takahiro Nawano, Ryosuke Tatsunami, Ko-ichi Ohtaki, Yoshiko Tampo, Nobumasa Hayase

**Affiliations:** 1 Department of Pharmacology & Therapeutics, Hokkaido Pharmaceutical University School of Pharmacy, 7-15-4-1 Maeda, Teine, Sapporo, Hokkaido 006-8590 Japan; 2 Department of Hospital Pharmacy & Pharmacology, Asahikawa Medical University, Asahikawa, 078-8510 Japan; 3 Department of Pharmacy, Shin-Sapporo Towakai Hospital, Sapporo, 004-0041 Japan; 4 Department of Dispensary, Rainbow Community Pharmacy, Sapporo, 062-0012 Japan; 5 Department of Public & Health, Hokkaido Pharmaceutical University School of Pharmacy, Sapporo, 006-8590 Japan

**Keywords:** Clarithromycin, Anti-inflammatory effect, Low-dose, Long-term treatment, Human small airway epithelial cells, Interleukin-8, Nuclear factor-κB, Nuclear factor erythroid 2-related factor 2, Oxidant/antioxidant balance, γ-Glutamylcysteine synthetase

## Abstract

**Background:**

Clarithromycin (CAM), a representative macrolide antibiotic, has been used widely at low doses for long-term therapy of chronic inflammatory airway diseases. Anti-inflammatory effects of macrolide antibiotics were first discovered in clinical practice. Although oxidative stress is known as a key pathogenesis factor in chronic airway inflammatory diseases, the mechanism of action of low-dose, long-term CAM therapy remains unclear. We aimed to examine the cytoprotective action of CAM against hydrogen peroxide (H_2_O_2_)-induced cell dysfunction, focusing on CAM dose and treatment duration, and using human small airway epithelial cells (SAECs), the main cells involved in chronic airway inflammatory diseases.

**Methods:**

SAECs were pretreated with CAM (1, 5 or 10 μM) for 24, 48 or 72 h, and were subsequently exposed to H_2_O_2_ for 0.5–4 h. Levels of interleukin (IL)-8, glutathione (GSH) and glutathione disulfide (GSSG), and the activities of nuclear factor (NF)-κB and γ-glutamylcysteine synthetase (γ-GCS) were assayed using specific methods. IL-8 mRNA and NF erythroid 2-related factor 2 (Nrf2) mRNA expression were measured using real-time reverse transcription polymerase chain reaction (RT-PCR). Tukey’s multiple comparison test was used for analysis of statistical significance.

**Results:**

Pretreatment with low-dose (1 or 5 μM), long-term (72 h) CAM inhibited H_2_O_2_-induced IL-8 levels, NF-κB activity, and IL-8 mRNA expression, and improved the GSH/GSSG ratio via the maintenance of γ-GCS expression levels. Similar to its enhancing effect on the GSH/GSSG ratio, pretreatment with low-dose CAM for 72 h significantly increased Nrf2 mRNA expression (*p* < 0.01 and *p* < 0.05). In contrast, these alterations were not observed after pretreatment with high-dose (10 μM) or short-term (24 and 48 h) CAM.

**Conclusions:**

CAM is efficacious against cell dysfunction caused by oxidative stress under low-dose, long-term treatment conditions. This effect depended on the suppression of NF-κB activation and improvement of the H_2_O_2_-induced oxidant/antioxidant imbalance that is achieved by increasing Nrf2 mRNA expression in SAECs. The present study may provide the first evidence of why low-dose, long-term administration of macrolides is effective for treating chronic inflammatory airway diseases.

**Electronic supplementary material:**

The online version of this article (doi:10.1186/s40360-017-0119-8) contains supplementary material, which is available to authorized users.

## Background

Macrolides such as clarithromycin (CAM) have been reported to be effective for the treatment of chronic inflammatory airway diseases at low doses and with long-term administration [1–5]. The effectiveness of macrolides in treating inflammatory airway diseases has been thought to be due to immunomodulatory effects rather than to direct antimicrobial activity. For instance, both CAM and erythromycin (EM) inhibit the production of inflammatory cytokines, such as interleukin (IL)-6 and IL-8, inhibit the release of soluble intracellular adhesion molecule (ICAM)-1 from airway epithelial cells, and decrease airway neutrophil accumulation [6, 7]. However, there are limited data concerning the potential benefits of low-dose, long-term application of CAM in a variety of chronic inflammatory airway diseases.

Environment-derived reactive oxygen species (ROS) are a primary cause of airway inflammation. Cigarette smoking has been proven to be the most important risk factor for causing airway oxidative stress [8]. In addition to external causes, the ROS hydrogen peroxide (H_2_O_2_) is known to increase chronic airway inflammation [9–13]. IL-8 is a neutrophil chemoattractant peptide, and it can induce polymorphonuclear neutrophil oxidative bursts that release H_2_O_2_ into the airways and lungs [14]. Furthermore, production of IL-8 is mediated by the activation of transcription factors such as nuclear factor (NF)-κB. Exposure to a number of stimuli, including ROS, results in the activation of NF-κB target genes including IL-8, and leads to increased synthesis of inflammatory proteins in the lung [15, 16]. Similarly, H_2_O_2_ induces glutathione (GSH, L-glutamyl-L-cysteinylglycine) production via NF-κB activation in bronchial epithelial and endothelial cells [17–19]. GSH is known to play an important role in the modulation of the oxidant/antioxidant balance in bronchial epithelium. Thus, GSH clears ROS and protects the bronchial epithelium from oxidative damage. GSH is produced via the activity of γ-glutamylcysteine synthetase (γ-GCS), an enzyme that catalyzes the first step and the rate-limiting step in de novo GSH synthesis [20]. Nuclear factor erythroid 2-related factor 2 (Nrf2) is a transcription factor that plays a key role in the regulation of γ-GCS expression and activity. Nrf2 is usually in a resting state in the cytoplasm. However, when it is stimulated by stresses such as oxidative stress, Nrf2 moves into the nucleus and binds to DNA. This binding then leads to the expression of various cytoprotective genes including γ-GCS [21]. Nrf2 thereby maintains intracellular GSH levels and redox homeostasis and plays an important role in protecting cells against oxidative damage.

Several reports have described the effect of macrolides on ROS-induced pulmonary inflammation or airway epithelial cell damage. Mikura et al. [22] and Zhou et al. [23] demonstrated that erythromycin (EM) prevents the lung inflammation induced by exposure to cigarette smoke in mice and rats. Kobayashi et al. [24] demonstrated that solithromycin (a novel macrolide), CAM, azithromycin, and telithromycin are protective against cigarette smoke-induced neutrophilia and IL-8 production in bronchoalveolar lavage fluid in mice. In addition to these in vivo experiments, it has been reported that the increases in IL-8 and GSH levels induced by H_2_O_2_ were abrogated by preincubation of human bronchial epithelial cells with EM [25]. Moreover, CAM significantly reduced soluble ICAM-1 expression induced by lipopolysaccharide (LPS)-induced oxidative stress in cultured rat tracheal epithelial cells [26]. Nevertheless, it is believed that these results, which show a concentration-dependent inhibitory effect after a relatively short incubation time, are not consistent with the effect of long-term clinical administration of macrolides at low doses.

In addition to the above data, CAM was observed to inhibit NF-κB activation in human peripheral blood mononuclear and pulmonary epithelial cells [27]. Furthermore, CAM suppressed IL-8 production via inhibition of NF-κB in human monocytes [28], while EM also inhibited GSH production and γ-GCS expression via inhibition of NF-κB in bronchial epithelial cells [25]. However, another report showed that CAM did not suppress NF-κB activation in human bronchial epithelial cells [29]. Thus, the inhibitory effects of macrolides on NF-κB activation are controversial. On the other hand, it is also known that Nrf2 controls not only γ-GCS gene expression but also NF-κB activation [30, 31]. However, there are no reports describing direct effects of macrolides on Nrf2 activity.

The present study was designed to answer the following questions: 1) are concentration and incubation time important factors in mediating the cytoprotective action of macrolides against H_2_O_2_-induced cell dysfunction; 2) do macrolide antimicrobial agents inhibit H_2_O_2_-induced oxidant/antioxidant imbalance and NF-κB activation; and 3) is Nrf2 activation involved in the effects of macrolides on GSH synthesis? Human small airway epithelial cells (SAECs), which are the main cell type involved in chronic inflammatory airway diseases, were used as model cells. Furthermore, CAM was used as a representative macrolide because its clinical efficacy against respiratory inflammatory disease is relatively high compared to that of other macrolides [32].

## Methods

### Chemicals and reagents

CAM, H_2_O_2_ (30%), the WST-8 (2-(2-methoxy-4-nitrophenyl)-3-(4-nitrophenyl)-5-(2,4- disulfophenyl)-2H-tetrazolium) assay system, GSH, and 5,5’-dithiobis(2-nitrobenzoic acid) (DTNB) were purchased from Wako Pure Chemical Industries, Ltd. (Osaka, Japan). GSH reductase (from yeast) and reduced nicotinamide adenine dinucleotide phosphate (NADPH) were from Oriental Yeast Co., Ltd. (Tokyo, Japan). Buthionine sulfoximine (BSO) and mouse anti-β-actin monoclonal antibody were from Sigma Chemical Co. (St. Louis, MO). A human IL-8 enzyme-linked immunosorbent assay (ELISA) kit was purchased from Bender Med Systems (Vienna, Austria). Rabbit polyclonal antibody against γ-GCS was from Neomarkers (Fremont, CA). The nuclear extraction and nuclear transcription factor ELISA kits were from Panomics (Fremont, CA). Cell culture media were obtained from Lonza (Walkersville, MD). All other chemicals used were of reagent grade.

### Cell culture and treatment

SAECs, cells of a normal human small airway epithelial cell line, were purchased from Lonza. SAECs were cultured in small airway growth medium (SAGM) supplemented with 0.1% bovine pituitary extract, 0.1% hydrocortisone, 0.1% insulin, 30 μg/mL gentamicin (GM), 15 ng/mL amphotericin B (AMTB), 0.1% fatty acid-free bovine serum albumin, 0.1% retinoic acid, 0.1% transferrin, 0.1% triiodothyronine, 0.1% epinephrine, and 0.1% human recombinant epidermal growth factor. Cells were seeded in 75-cm^2^ filter vent flasks (Corning, NY) and grown to 80% confluence (3 × 10^6^ cells/well) for each experimental condition at 37 °C, in a humidified atmosphere of 5% CO_2_ and 95% air. The culture medium was changed on day 1 and subsequently every 2 days. Cells were passaged by trypsinization, and cultures between passages 3 and 9 were used for all experiments. Cells were cultured in the presence or absence of CAM (1, 5 or 10 μM) for 24–72 h and were then stimulated with H_2_O_2_ (100 μM) for an additional 0.5–4 h. Pretreatments with CAM were carried out with a different set of cells for each concentration and treatment duration. In addition, cell stimulation with H_2_O_2_ was performed as follows in order to determine at which time the effect of H_2_O_2_ treatment was the strongest. For stimulation of the cells with H_2_O_2_, the medium was changed to small airway basal medium (SABM) containing no supplements because bovine pituitary extract and serum may include antioxidants, chelators of transition metal ions, and high-density lipoproteins [33]. CAM was dissolved in dimethylsulfoxide at a final concentration of 10 mM, and was then diluted with distilled water or culture medium to the desired concentrations. The final dimethylsulfoxide concentration was less than 0.1%. CAM solution used for experiments was prepared immediately before use.

### Cell proliferation and viability

SAECs (3 × 10^4^ cells/well) seeded in collagen-coated 96-well plates were treated with CAM (1, 5, 10 or 30 μM) for 24, 48 or 72 h or with H_2_O_2_ (100, 300 or 500 μM) for 24 h. Post-treatment, the SAEC medium was changed to basal medium containing 10% WST-8 solution and the cells were incubated at 37 °C for 2 h. The WST-8 solution is reduced by living cells to give an orange-colored formazan product with an absorbance at 450 nm. The optical density (OD) of the culture medium was measured at 450 nm with a spectrophotometric microliter plate reader (Bio-Rad; Hercules, CA) at 24, 48, and 72 h after treatment. Cell proliferation and viability are expressed as the ratio of surviving cells to untreated cells. The morphology of SAECs was evaluated visually throughout the course of the experiments.

### IL-8 protein levels in culture supernatants

SAECs (3 × 10^4^ cells/well) in 96-well plates were pretreated with CAM (1, 5 or 10 μM) for 24, 48 or 72 h and were then stimulated with H_2_O_2_ (100 μM) for 4 h. Culture media supernatants were collected and centrifuged to remove cell debris (12,000 × *g* for 10 min). The supernatants were frozen at -80 °C until use in assays. IL-8 protein levels in the culture supernatants were analyzed using ELISA according to the manufacturer’s instructions. Briefly, experimental samples were added into individual wells coated with human monoclonal antibody specific for IL-8 and were incubated for 3 h at room temperature. After six washes with phosphate-buffered saline (PBS) containing 0.1% Tween 20 to remove unbound protein, tetramethyl-benzidine was added to each well and incubated for 10 min at room temperature. The reaction was terminated by the addition of 1 M phosphoric acid. The color generated in each sample was determined by measurement of OD at 450 nm using a spectrophotometric microliter plate reader. The IL-8 protein levels of unknown samples were calculated using a standard curve. Data are expressed as means ± SD in pg/ml culture supernatants. This assay was specific for human IL-8 and the antibody did not cross-react with IL-6, IL-2R, recombinant human lymphotoxin (rhTNF*β*), or CD8 antigen. Assay sensitivity was 1.3 pg/ml and intra- and inter-assay variations were less than 8.1%.

### IL-8 mRNA expression

SAECs (10^6^ cells/well) in 6-well plates were pretreated with CAM (1, 5 or 10 μM) for 24, 48, or 72 h and were then stimulated with H_2_O_2_ (100 μM) for 1 h. Total RNA was obtained using a PureLink RNA Mini Kit (Life Technologies Corp., Carlsbad, CA, USA) following the manufacturer’s instructions and was quantified by measurement of absorbance at 260 nm. RNA (2 μg) was reverse transcribed into complementary deoxyribonucleic acid (cDNA) using a SuperScript VILO cDNA Synthesis Kit following the manufacturer’s instructions. TaqMan polymerase chain reaction (PCR) primers and probes for IL-8 and for glyceraldehyde-3-phosphate dehydrogenase (GAPDH) as the internal standard gene were purchased from Applied Biosystems (Foster City, CA). TaqMan PCR was performed with 1 μl of sample cDNA in a 20-μl reaction mixture containing TaqMan gene master mix and TaqMan gene expression assay. Amplification was performed using the 7500 Real Time Reverse Transcription-PCR System (Applied Biosystems). The PCR thermal protocol consisted of 50 °C for 2 min and 95 °C for 10 min, followed by 40-cycle amplification at 95 °C for 15 s and 60 °C for 1 min. Relative quantification of gene expression was performed using the comparative threshold method. Changes in mRNA expression were calculated after normalizing to GAPDH, and are expressed as a ratio to changes in untreated cells.

### Extraction of nuclear proteins and transcription factor assay

SAECs (10^6^ cells/well) in 6-well plates were pretreated with CAM (1, 5 or 10 μM) for 24, 48 or 72 h and were then stimulated with H_2_O_2_ (100 μM) for 1 h. Cells were washed with ice-cold PBS and nuclear extracts were obtained using a nuclear extraction kit according to the manufacturer’s instructions (Panomics Co. Fremont, CA). Nuclear protein was measured using the DC protein assay (Bio-Rad) and a 10 μg aliquot of nuclear protein was applied to a Transcription Factor ELISA kit to assay NF-κB.

### Western blot analysis for IκBα phosphorylation and γ-GCS protein

Phosphorylated inhibitor κBα (p-IκBα) and γ-GCS protein levels were analyzed by western blotting. SAECs (2 × 10^6^ cells/well) in 6-cm dishes were pretreated with CAM (1, 5 or 10 μM) for 72 h and were then stimulated with H_2_O_2_ (100 μM) for 0.5 h for p-IκBα and 1 h for γ-GCS analysis. Cells were washed with Dulbecco’s phosphate-buffered saline (DPBS) and were collected using fresh DPBS and a cell scraper. After centrifugation at 2000 × *g* for 10 min, 50 μl of radioimmunoprecipitation assay buffer (Pierce, Rockford, IL) containing 1 M vanadate and protease inhibitors was added and then cell pellets were sonicated three times for 10 s each time. The lysates were centrifuged at 12,000 × *g* for 10 min and then an aliquot of the supernatant containing 10 μg of protein was resuspended in an equal amount of sample buffer (Laemmli sample buffer containing 0.5 mM of 2-mercaptoethanol) and was boiled for 5 min. Protein concentration was assayed using the same method as that used for the GSH and GSSG assays that are described in the following experiment. Samples that had been refrigerated were separated by 12% sodium dodecyl sulfate polyacrylamide gel electrophoresis, and the gel was then electrotransferred onto a nitrocellulose membrane (Bio-Rad). Membranes were blocked in 5% nonfat dry milk in a Tris-buffered solution containing 0.1% Tween 20 at room temperature for 2 h. The membrane was incubated with primary antibodies for p-IκBα, γ-GCS or β-actin with horseradish peroxidase-conjugated secondary antibodies. Signal was detected as the intensity of chemiluminescence using an ECL Plus Western Blotting Detection Kit (GE Healthcare, Buckinghamshire, UK). p-IκBα or γ-GCS levels were normalized to constitutive expression of β-actin protein, and are expressed as p-IκBα/β**-**actin or γ-GCS/β-actin calculated as the scan unit ratio (%) ± SD of four experiments.

### Measurement of GSH and GSSG levels

Intracellular GSH and GSSG levels were measured using the DTNB recycling method [34]. SAECs (5 × 10^5^ cells/well) in 12-well plates were pretreated with CAM (1, 5 or 10 μM) for 72 h and were then stimulated with H_2_O_2_ (100 μM) for 2 h. The cells were washed with DPBS and solubilized with 220 μl of PBS containing 0.1% Triton X-100. After centrifugation, cell supernatants were collected and used as the total GSH (GSH and GSSG) sample. To obtain the GSSG sample, an aliquot of the supernatant (100 μl) was mixed with 2-vinylpyridine (2.4 μl) and the mixture was adjusted to pH 6.8 with 0.1 M sulfuric acid (4.8 μl). The solution was mixed vigorously for 1 min and incubated at 25 °C for 20 min. Each sample was then used for total GSH or GSSG assay by adding 0.2 mM NADPH, 0.6 mM DTNB and GSH reductase (1.3 U/ml), and the 2-nitro-5-thiobenzoic acid produced by the samples was measured spectrophotometrically (Hitachi Co., Tokyo, Japan) at 412 nm for 5 min. GSH was calculated by determination of the difference between total GSH and GSSG levels. Protein concentration was determined using the Bradford method with bovine serum albumin as the standard. Data are expressed as the GSH/GSSG ratio ± SD of four experiments.

### Nrf2 mRNA expression

Nrf2 mRNA expression in SAECs was examined to determine if CAM directly affects Nrf2 activity. SAECs (10^6^ cells/well) in 6-well plates were pretreated with CAM (1, 5 or 10 μM) for 72 h and were then stimulated with H_2_O_2_ (100 μM) for 1 h. Nrf2 mRNA expression was measured using the same method as that used for measurement of IL-8 mRNA expression, except that TaqMan PCR primers and probes for Nrf2 instead of for IL-8 were used.

### Cell viability following BSO treatment

SAECs (3 × 10^4^ cells/well) in 96-well plates were pretreated with BSO (1 mM) for 16 h and were then stimulated with H_2_O_2_ (100 μM) for 1, 3 or 6 h. For comparison, cells were pretreated with CAM (1, 5 or 10 μM) for 72 h and were then stimulated with H_2_O_2_ (100 μM) for 3 h. After the H_2_O_2_ treatment, cell viability was evaluated using the WST-8 assay method as described above.

### Measurement of intracellular ROS levels

Intracellular ROS levels were measured using flow cytometry with 5 (and 6)-carboxy-2’ , 7’-dichlorofluorescein diacetate (CDF) as a fluorescent probe of intracellular ROS. SAECs (5 × 10^5^ cells/well) in 12-well plates were pretreated with CAM (1, 5 or 10 μM) for 72 h and were then treated with CDF (1 μM) for 0.25 h before stimulation with H_2_O_2_ (100 μM) for 0.25 h. After the H_2_O_2_ treatment, the cells were washed with DPBS and were then filtered through a nylon mesh (35 μm). The fluorescence intensity of CDF in the obtained sample was measured at 488 nm (excitation wavelength) and 530 nm (fluorescence wavelength) using a flow cytometer (Beckman Coulter Inc., Brea, CA).

### Measurements of extra- and intracellular CAM concentrations using HPLC

To evaluate CAM concentrations in culture supernatants and within cells, SAECs were cultured for 24, 48 or 72 h with CAM. Cell treatments and sample preparation were performed according to the method described by Togami et al. [35]. Briefly, SAECs were suspended in SAGM at a density of 10^6^ cells/ml. The cell suspension was seeded into 96-well plates and incubated for 48 h. After this incubation, the cells were treated with CAM (1, 5 or 10 μM) for 24, 48 or 72 h. SAECs were collected by centrifugation. The cell pellets were washed three times with ice-cold PBS to remove any unbound CAM and were then solubilized with a solution of 0.1 M NaOH. These cell lysates (equivalent of 10^6^ cells/ml) were used as intracellular samples. The supernatants were collected into microtubes and were used as extracellular samples. These samples were stored at -80 °C. CAM concentration in the supernatants and cell lysates was measured using ion-paired high performance liquid chromatography (HPLC) as follows. A 50 μl aliquot of each cell supernatant or cell lysate was mixed with ethyl 4-aminobenzoate as an internal standard, which was dissolved in acetonitrile (50 μl), and the mixture was vortexed vigorously. After centrifugation at 12,000 × g for 10 min, a 50-μl aliquot of the supernatant was analyzed by HPLC (Jasco Co., Tokyo, Japan) with a Mightysil RP-18GP column (4.6 × 250 mm, 5 μm pore size; Kanto Chemical Co., Tokyo, Japan). The mobile phase consisted of 5 mM phosphate buffer (pH 3.5) and acetonitrile (55:45) containing 5 mM 1-octanesulfonic acid as an ion-paired reagent. The separation was performed at a flow rate of 1.0 ml/min at 50 °C and was monitored at 210 nm using an ultraviolet detector.

### Statistical analysis

All data are expressed as means ± standard deviation (SD). Statistical analysis was performed using one-way analysis of variance (ANOVA), and differences, which were estimated by Tukey’s multiple comparison test after the Shapiro-Wilk test and Bartlett’s test, were considered to be statistically significant at *p* < 0.05.

## Results

### Effects of CAM and H_2_O_2_ on SAEC cell growth

**Fig. 1:**
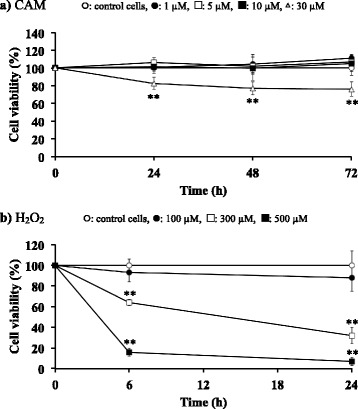
Effects of CAM (**a**) and H_2_O_2_ (**b**) on cell viability in SAECs. Cells were incubated with 1 μM, 5 μM, 10 μM or 30 μM CAM or with 100 μM, 300 μM, or 500 μM H_2_O_2_ for 72 or 24 h, respectively. Cell viability was assessed by measuring formazan production from viable cells (at 450 nm) as described in the Materials and Methods. Data presented in panels **a** and **b** are means ± SD of six independent experiments. **p < 0.01 vs. control cells

The WST-8 assay demonstrated that neither 1, 5 or 10 μM of CAM nor 100 μM of H_2_O_2_ affected SAEC proliferation or viability. No morphological changes were visible microscopically in either resting SAECs or in these CAM- or H_2_O_2_-treated cells. However, 30 μM of CAM significantly inhibited the viability of SAECs at all time points assayed (Fig. 1a and see Additional file 1, *p* < 0.01 vs. control), and both 300 and 500 μM of H_2_O_2_ significantly induced cell death at both 6 and 24 h after treatment (Fig. 1b and see Additional file 2, *p* < 0.01 vs. control). Based on these data, in the following experiments, H_2_O_2_ was used at a concentration of 100 μM, and CAM was used at concentrations of 1 or 5 μM (low) or 10 μM (high).

### Effects of CAM pretreatment on H_2_O_2_-induced IL-8 protein release in SAECs

**Fig. 2:**
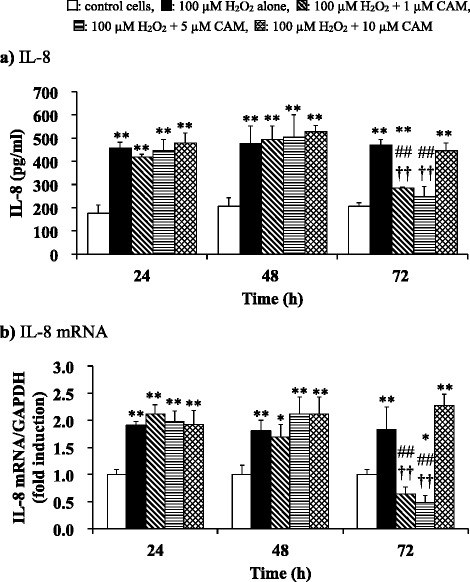
Effects of CAM pretreatment on IL-8 protein (**a**) and mRNA (**b**) levels in H_2_O_2_-stimulated SAECs. In panel **a**, levels of IL-8 protein were measured using the ELISA method. In panel **b**, IL-8 mRNA expression was measured using real-time RT-PCR. Samples were obtained from supernatants (**a**) or cDNA (**b**) of control cells, of cells stimulated with 100 μM H_2_O_2_ alone, or of cells pretreated with 1 μM, 5 μM or 10 μM CAM for 24, 48 or 72 h before stimulation with 100 μM H_2_O_2_ for 4 or 1 h, respectively. Data are presented as means ± SD of four independent experiments. *p < 0.05, **p < 0.01 vs. control cells, ^##^ *p* < 0.01 vs. cells stimulated with H_2_O_2_ alone, ^††^ *p* < 0.01 vs. cells pretreated with 10 μM CAM

Normally cultured SAECs released low levels of IL-8 protein into the culture medium. The exposure of SAECs to H_2_O_2_ (100 μM) caused a dramatic increase in supernatant IL-8 levels after 4 h. Pretreatment of the SAECs with low or high concentrations of CAM for up to 48 h prior to H_2_O_2_ treatment had no influence on H_2_O_2_–induced IL-8 protein secretion. However, pretreatment with 1 or 5 μM CAM, but not with 10 μM CAM, for 72 h prior to H_2_O_2_ treatment, significantly decreased H_2_O_2_-induced IL-8 protein release (*p* < 0.01) (Fig. 2a and see Additional file 3). There was a significant difference in secreted IL-8 protein level between the low- and high-concentration CAM groups (*p* < 0.01). These results showed that low-dose, long-term CAM treatment could effectively suppress H_2_O_2_-induced IL-8 protein release in SAECs.

### Effects of CAM pretreatment on H_2_O_2_-induced IL-8 mRNA expression in SAECs

IL-8 mRNA expression in SAECs was significantly increased by H_2_O_2_ treatment (100 μM) for 1 h. Similar to its effects on H_2_O_2_-induced IL-8 release, pretreatment with low or high concentrations of CAM for up to 48 h had no effect on H_2_O_2_-induced IL-8 mRNA expression. Notably, pretreatment with 1 or 5 μM CAM, but not with 10 μM CAM, for 72 h significantly suppressed this H_2_O_2_-induced IL-8 mRNA expression compared to H_2_O_2_ treatment alone (*p* < 0.01) (Fig. 2b and see Additional file 4). Again, similar to the effect of CAM on H_2_O_2_-induced IL-8 release, there was a significant difference in H_2_O_2_-induced IL-8 mRNA expression between the low- and high-concentration CAM groups (*p* < 0.01).

### Effects of CAM pretreatment on H_2_O_2_-induced NF-κB activation in SAECs

**Fig. 3:**
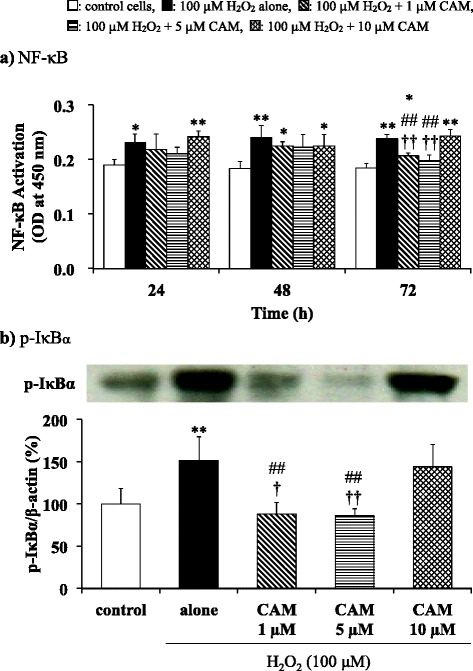
Effects of CAM pretreatment on NF-κB (**a**) and p-IκBα (**b**) activation in SAECs stimulated with H_2_O_2_. In panel **a**, NF-κB activities were measured using an ELISA method. In panel **b**, p-IκBα protein levels were detected by western blotting (upper, representative blot images; lower, quantification of bands). Samples were obtained from nuclear proteins of control cells, of cells stimulated with 100 μM H_2_O_2_ alone, or of cells pretreated with 1 μM, 5 μM or 10 μM CAM for 24, 48 or 72 h before stimulation with 100 μM H_2_O_2_ for 0.5–1 h. The data in panel **b** are of cells preincubated with CAM for 72 h prior to H_2_O_2_ stimulation, and are expressed as the p-IκBα/β-actin ratio. Data are presented as means ± SD of four independent experiments. *p < 0.05, **p < 0.01 vs. control cells, ^##^ *p* < 0.01 vs. cells stimulated with H_2_O_2_ alone, ^†^ *p* < 0.05, ^††^ *p* < 0.01 vs. cells pretreated with 10 μM CAM

Using a specific oligonucleotide probe for NF-κB binding sites in a transcription factor activity ELISA assay, the nuclear extracts from resting SAECs showed NF-κB binding activity. This binding activity in SAECs was significantly increased by treatment with H_2_O_2_ (100 μM) for 1 h. Pretreatment with CAM for up to 48 h had no influence on this H_2_O_2_-induced NF-κB binding activity. However, after 72 h pretreatment with 1 or 5 μM CAM, but not with 10 μM CAM, H_2_O_2_-induced activation of NF-κB was significantly decreased (*p* < 0.01) (Fig. 3a and see Additional file 5). There was also a significant difference in H_2_O_2_-induced NF-κB binding activity between the low- and high-concentration CAM groups (*p* < 0.01).

The above experimental results indicated that pretreatment with CAM for up to 48 h had no influence on H_2_O_2_-induced cellular disarrangements. Therefore, in the following experiments, the effects of CAM on H_2_O_2_-induced alterations in SAECs were examined following CAM pretreatment for 72 h.

### Effects of CAM on H_2_O_2_-induced IκBα phosphorylation in SAECs

IκBα phosphorylation was significantly increased by H_2_O_2_ treatment (100 μM) for 0.5 h as assessed by western blotting. Similar to its effects on NF-κB activation, pretreatment with 1 or 5 μM CAM for 72 h significantly suppressed this H_2_O_2_-induced phosphorylation compared to H_2_O_2_ treatment alone (*p* < 0.01), whereas pretreatment with 10 μM CAM did not (Fig. 3b and see Additional file 6). There was a significant difference in H_2_O_2_-induced phosphorylation between the low- and high-concentration CAM groups (*p* < 0.05 or *p* < 0.01, respectively), similar to their effects on NF-κB activation.

### Effects of CAM pretreatment on the H_2_O_2_-induced GSH/GSSG ratio in SAECs

**Fig. 4:**
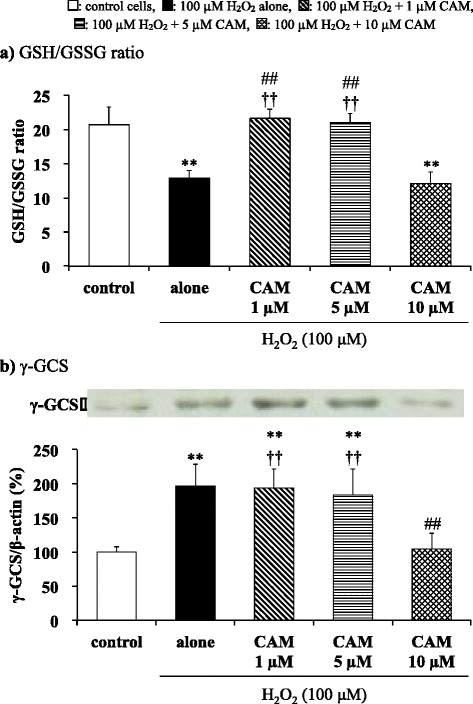
Effects of CAM pretreatment on the GSH/GSSG ratio (**a**) and γ-GCS expression (**b**) in H_2_O_2_-stimulated SAECs. In panel **a**, GSH and GSSG were determined using the DTNB recycling method. In panel **b**, γ-GCS expression was detected by western blotting (upper, representative blot images; lower, quantification). Samples were obtained from supernatants (**a**) or cell pellets (**b**) of control cells, of cells stimulated with 100 μM H_2_O_2_ alone, or of cells pretreated with 1 μM, 5 μM or 10 μM CAM for 72 h before stimulation with 100 μM H_2_O_2_ for 1–2 h. The data in panel **b** are expressed as the γ-GCS/β-actin ratio. Data are presented as means ± SD of four independent experiments. **p < 0.01 vs. control cells, ^##^ *p* < 0.01 vs. cells stimulated with H_2_O_2_ alone, ^††^ *p* < 0.01 vs. cells pretreated with 10 μM CAM

Incubation with H_2_O_2_ for 2 h significantly decreased the GSH/GSSG ratio in SAECs compared to untreated cells (*p* < 0.01). Pretreatment with a low concentration (1 or 5 μM), but not with a high concentration of CAM (10 μM) for 72 h significantly increased this ratio in H_2_O_2_ treated cells (Fig. 4a and see Additional file 7, *p* < 0.01 vs. H_2_O_2_ treatment alone). There was a significant difference in the H_2_O_2_-induced GSH/GSSG ratio between the low- and high-concentration CAM groups (*p* < 0.01). Furthermore, these effects of CAM on the H_2_O_2_-induced GSH/GSSG ratio are the inverse of the effects of CAM on H_2_O_2_-induced IL-8 protein release or IL-8 mRNA expression in SAECs as described above.

### Effects of CAM pretreatment on H_2_O_2_-induced γ-GCS protein expression in SAECs

The effects of CAM on H_2_O_2_-induced γ-GCS protein expression in SAECs was investigated using western blotting. Exposure of SAECs to H_2_O_2_ (100 μM) significantly increased γ-GCS protein expression versus control cells, and this increased γ-GCS expression was maintained with treatment with 1 or 5 μM CAM for 72 h before H_2_O_2_ treatment. However, pretreatment with 10 μM CAM for 72 h resulted in a significant decline in H_2_O_2_-induced γ-GCS expression (Fig. 4b and see Additional file 8, *p* < 0.01 vs. H_2_O_2_ treatment alone). As expected, pretreatment with a high concentration (10 μM) of CAM for 72 h also significantly decreased H_2_O_2_-induced γ-GCS levels compared to pretreatment with a low concentration (1 or 5 μM) of CAM for 72 h (*p* < 0.01).

### Effects of CAM pretreatment on H_2_O_2_-induced Nrf2 mRNA expression in SAECs

**Fig. 5:**
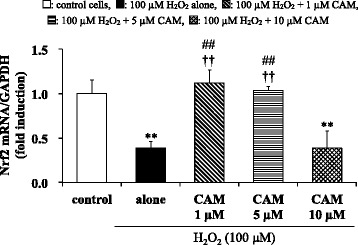
Effects of CAM on Nrf2 mRNA expression in SAECs stimulated with H_2_O_2_. Nrf2 mRNA was measured using real-time RT-PCR. Samples were obtained from cell pellets of control cells, of cells stimulated with 100 μM H_2_O_2_ alone, or of cells pretreated with 1 μM, 5 μM or 10 μM CAM for 72 h before stimulation with 100 μM H_2_O_2_ for 1 h. Data are presented as means ± SD of four independent experiments. **p < 0.01 vs. control cells, ^##^ *p* < 0.01 vs. cells stimulated with H_2_O_2_ alone, ^††^ *p* < 0.01 vs. cells pretreated with 10 μM CAM

Nrf2 mRNA expression was significantly suppressed by H_2_O_2_ treatment (100 μM) for 1 h. Similar to its effects on the GSH/GSSG ratio, pretreatment with 1 or 5 μM CAM, but not with 10 μM CAM, for 72 h significantly increased Nrf2 mRNA expression in H_2_O_2_ treated cells compared to H_2_O_2_ treatment alone (*p* < 0.01) (Fig. 5 and see Additional file 9). As expected, there was a significant difference in Nrf2 mRNA expression in H_2_O_2_ treated cells between the low- and high-concentration CAM groups (*p* < 0.01). These effects of CAM on H_2_O_2_-induced Nrf2 mRNA expression are the inverse of its effects on H_2_O_2_-induced NF**-**κB activation or IκBα phosphorylation in SAECs that are described above.

### Effects of BSO and CAM pretreatment on the viability of H_2_O_2_-treated cells

**Fig. 6:**
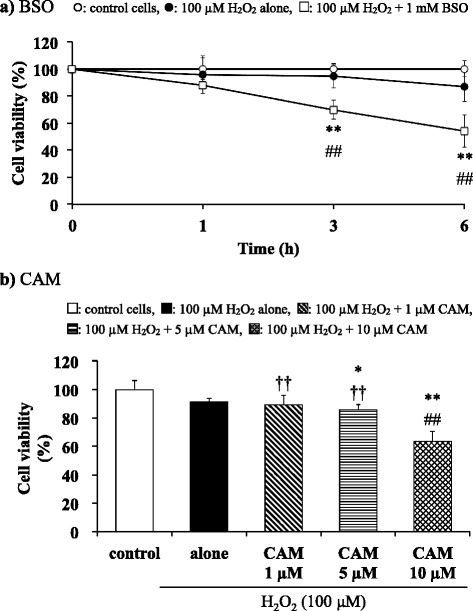
Effects of BSO (**a**) and CAM (**b**) on cell viability in SAECs stimulated with H_2_O_2_. In panel **a**, samples were obtained from control cells, or from cells pretreated with or without 1 mM BSO for 16 h before stimulation with 100 μM H_2_O_2_ for 1, 3 or 6 h. In panel **b**, samples were obtained from control cells, from cells stimulated with 100 μM H_2_O_2_ alone, or from cells pretreated with 1 μM, 5 μM or 10 μM CAM for 72 h before stimulation with 100 μM H_2_O_2_ for 3 h. Data are presented as means ± SD of six independent experiments. *p < 0.05, **p < 0.01 vs. control cells, ^##^ *p* < 0.01 vs. cells stimulated with H_2_O_2_ alone, ^††^ *p* < 0.01 vs. cells pretreated with 10 μM CAM

BSO inhibits γ-GCS to deplete cellular GSH. Pretreatment of SAECs with BSO for 3 or 6 h aggravated the loss of cell viability induced by treatment with 100 μM H_2_O_2_ (Fig. 6a and see Additional file 10). Similarly, pretreatment with 10 μM CAM for 72 h before H_2_O_2_ (100 μM) treatment caused a significant decrease in cell viability compared to H_2_O_2_ treatment alone (*p* < 0.01, Fig. 6b and see Additional file 11). In contrast, the viability of cells treated with H_2_O_2_ (100 μM) did not change when pretreated with a low concentration (1 or 5 μM) of CAM. The low- and high-concentration CAM groups differed significantly in their effects on the viability of H_2_O_2_ treated cells, similar to their effect on γ-GCS expression (*p* < 0.01, Fig. 6b and see Additional file 11).

### Intracellular ROS levels

**Fig. 7:**
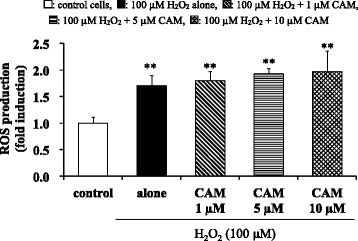
Effects of CAM on intracellular ROS levels in SAECs stimulated with H_2_O_2_. Intracellular ROS levels were measured using flow cytometry with the fluorescent probe CDF. Samples were obtained from control cells, from cells stimulated with 100 μM H_2_O_2_ alone, or from cells pretreated with 1 μM, 5 μM or 10 μM CAM for 72 h before stimulation with 100 μM H_2_O_2_ for 0.25 h. CDF (1 μM) was applied to cells for 0.25 h before stimulation with H_2_O_2_. Data are presented as means ± SD of four independent experiments. **p < 0.01 vs. control cells

We next examined whether H_2_O_2_ modulation of intracellular ROS levels was affected by pretreatment of the cells with CAM. Intracellular ROS production was significantly increased by H_2_O_2_ treatment (100 μM) for 0.25 h (*p* < 0.01 vs. control). However, pretreatment with CAM (1, 5 or 10 μM) for 72 h had no effect on the production of intracellular ROS compared to H_2_O_2_ treatment alone (Fig. 7 and see Additional file 12).

### Extra- and intracellular CAM concentrations

**Fig. 8:**
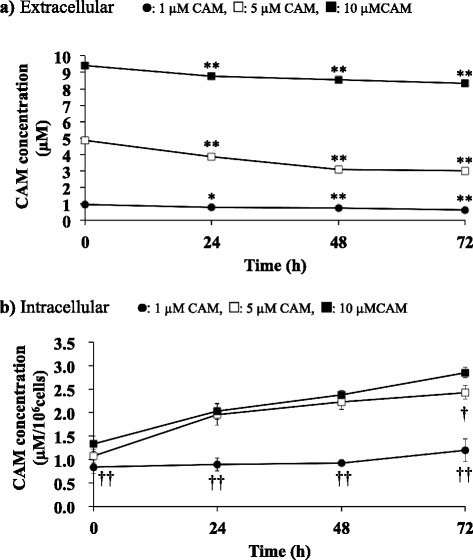
Time-dependent changes in extracellular (**a**) and intracellular (**b**) concentrations of CAM in SAECs. Cells were incubated with 1 μM, 5 μM or 10 μM CAM for 24, 48 or 72 h. CAM concentrations were measured using HPLC. Data are presented as means ± SD of four independent experiments. *p < 0.05, **p < 0.01 vs. cells incubated with the same concentration of CAM for 0 h. ^†^ *p* < 0.05, ^††^ *p* < 0.01 vs. cells incubated with 10 μM CAM at each time

We performed HPLC analysis of extracellular and intracellular levels of CAM to evaluate the stability of CAM in the culture medium and the amount of CAM that entered into the SAECs. Under the analytical conditions used, no peaks other than CAM were eluted on the chromatogram. The concentration of CAM in culture supernatants decreased in a time-dependent manner (Fig. 8a and see Additional file 13). Conversely, CAM levels in the cells increased in a time-dependent manner (Fig. 8b and see Additional file 14). The levels of intracellular CAM in the cells treated with 5 or 10 μM CAM were the same for up to 48 h of CAM treatment. However, after 72 h of treatment the intracellular CAM concentration in the cells treated with 10 μM CAM was significantly higher (*p* < 0.05) than that of the cells treated with 5 μM CAM. There was a significant difference in intracellular CAM concentration between the low- and high-concentration CAM groups after 72 h of treatment and between the 1 μM and 10 μM CAM groups (*p* < 0.01) at all time points.

## Discussion

The anti-inflammatory effects of macrolide antibiotics were first discovered in clinical practice [1], and much basic research has been subsequently reported [36]. However, the mechanism of action of low-dose, long-term therapy on airway inflammation remains unclear. Therefore, in this study, we examined the cytoprotective action of CAM against H_2_O_2_-induced cellular dysfunction (focusing on dose and duration) in SAECs, which are the main cell type involved in chronic inflammatory airway diseases.

First, we examined cell viability to identify sub-toxic concentrations for CAM and H_2_O_2_. Based on the results of those experiments, we used 1 and 5 μM CAM as the low-dose pretreatment, and 10 μM CAM as the high-dose pretreatment (Fig. 1). These CAM concentrations were deemed reasonable because an adult taking 200 mg of CAM per day (low dose) would exhibit CAM levels of approximately 1–5 μM in blood as well as in bronchial epithelial lining fluid [37], compared to approximately 11 μM with an intake of 400 mg per day (normal dose). Furthermore, during inflammatory conditions in the lower respiratory tract, neutrophil-derived H_2_O_2_ in respiratory tract viscous fluid is reported to reach a concentration of 50 μM [38]. Since H_2_O_2_ is also produced directly from bronchial epithelial cells following stimulation with pollutants such as bacteria and LPS [39], it is thought that the 100 μM H_2_O_2_ concentration used in this study reflects conditions observed during chronic inflammatory airway diseases.

Our study confirmed that CAM decreased H_2_O_2_-induced IL-8 protein release in SAECs (Fig. 2a). This decrease was associated with inhibited expression of IL-8 mRNA and suppression of transcription factor NF-κB activity (Figs. 2b and 3a). Among the cytokines reduced by macrolides, IL-8 was negatively regulated in most studies of airway epithelial cells, neutrophils, and monocytes [27–29]. In this study, however, it is clearly shown that CAM inhibited IL-8 synthesis after long-term treatment with a low dose, but not after a short-term treatment or treatment with a high dose. In addition, the phosphorylation of IκBα, which inactivates NF-κB in the cytoplasm, was also suppressed by long-term, low-dose CAM treatment, but not by a high CAM concentration (Fig. 3b). It is therefore thought that the inhibition of NF-κB activity is linked to inhibition of IκBα phosphorylation by CAM. There is a possibility that long-term, low-dose treatment with CAM has an effect on other inflammatory cytokines (such as IL-6) that are also regulated by NF-κB, similar to its effect on IL-8. Additionally, it was reported that the 14-OH metabolite of CAM strongly inhibited IL-8 production induced by tumor necrosis factor (TNF)-α in bronchial epithelial cells [40]. Although the 14-OH metabolite was not found in our HPLC analysis, it will be necessary to examine its influence on NF-κB activity in future studies.

In chronic inflammatory airway diseases, patients develop oxidant/antioxidant imbalance. The antioxidant GSH plays an important role in the development of oxidative stress tolerance. GSH is reduced by oxidation to the GSSG form, and reduction of the GSH/GSSG ratio causes chronic airway inflammation [41]. However, the effects of CAM on antioxidant status remain unclear. Rapid impairment of the GSH/GSSG ratio and rapid induction of intracellular GSH synthesis in SAECs were observed in response to H_2_O_2_-induced oxidative stress. These rapid changes may imply a critical determinant of cellular tolerance to oxidative stress [42]. Since SAECs are cells that directly contact the external environment, it is believed that these cells are more highly sensitive to oxidative stress compared to endothelial cells [43]. In this study, the GSH/GSSG ratio was significantly increased by long-term treatment with low CAM concentrations, which contributed to the improvement of oxidant/antioxidant imbalance (Fig. 4a). On the other hand, the expression of γ-GCS protein, a GSH synthesis enzyme, was significantly suppressed by treatment with long-term, high-dose CAM (Fig. 4b). It has been reported that H_2_O_2_-induced GSH production and γ-GCS expression in bronchial epithelial cells is suppressed by pretreatment with EM (5 μg/ml; ca. 6.8 μM) for 48 h, resulting in disturbance of the oxidant/antioxidant balance [25]. However, the effects of lower EM concentrations and longer incubation times were not examined. In contrast, our results indicated that long-term (72 h) treatment with a low CAM concentration (1 or 5 μM) increased γ-GCS expression to improve the oxidant/antioxidant imbalance. Nevertheless, the reason why γ-GCS expression was suppressed by treatment with a high CAM concentration (10 μM) for a long duration remains to be elucidated. Although γ-GCS expression is not accompanied by changes in NF-κB activity [44], the change in the GSH/GSSG ratio that is brought about by low CAM concentrations in this study appears to be in the inverse direction to the change brought about in NF-κB activation or IκBα phosphorylation. ROS causes increased gene expression of both proinflammatory genes and the protective gene γ-GCS by oxidant-mediated activation of transcription factors. Other transcription factors connected with NF-κB activation might be related to GSH production during oxidative stress [45]. Nrf2 is an important transcription factor that regulates GSH levels by controlling the expression of the γ-GCS gene [21]. Moreover, it is also reported that Nrf2 inhibited NF-κB activation in response to Nrf2 activities in vascular endothelial cells [30, 31]. Indeed, consistent with these reports, we clearly demonstrated that Nrf2 mRNA expression was increased to suppress NF-κB activation at low concentrations of CAM but not at a high concentration of CAM (Fig. 5). An increase in Nrf2 expression stimulates γ-GCS gene expression to increase the intracellular GSH level. Accordingly, it is considered that γ-GCS expression was suppressed in the case of treatment with a high CAM concentration for a long duration (Fig. 4b). However, for better understanding of the involvement of Nrf2 in GSH production after treatment with CAM, the effects of using knock-down and inhibitors of Nrf2 need to be examined in more detail in future studies. The culture medium used in this study contained GM (30 μg/mL) and AMTB (15 ng/mL) to prevent microbial contamination. Both GM and AMTB have been shown to induce changes in oxidant/antioxidant imbalance at concentrations of 10 mg/mL and 0.7–1.9 μg/mL, respectively, in vivo [46, 47]. However, since the concentrations of these agents in the medium used in the experiments of this study were about 50- to 300-fold lower than the values previously reported to modulate the oxidant/antioxidant balance, it is considered that the addition of these agents to the medium did not affect the oxidant/antioxidant balance in our experiments.

We further explored the relationship between γ-GCS activity and cell viability to determine the influence of γ-GCS suppression following long-term pretreatment with a high CAM concentration. Exposure to BSO resulted in increased H_2_O_2_-induced cytotoxicity in SAECs (Fig. 6a). This result indicates that cell viability is decreased when γ-GCS synthesis is suppressed during oxidative stress. This result means that intracellular GSH plays a critical role during H_2_O_2_-induced cytotoxicity in SAECs. However, in contrast to the effect of low-dose CAM, H_2_O_2_-induced cytotoxicity was not prevented by long-term pretreatment of SAECs with a high CAM concentration (Fig. 6b). Long-term pretreatment with a high CAM concentration may induce a disturbance similar to that induced by BSO in SAECs during oxidative stress.

CAM was not shown to have a direct radical scavenger activity against ROS (Fig. 7). Thus, low concentrations (1 and 5 μM) of CAM do not directly inhibit intracellular ROS, but instead indirectly inhibit the cell damage caused by ROS by activating a system that modulates the damaging effects of H_2_O_2_ by improving the GSH/GSSG ratio. Nevertheless, the effects of CAM are not observed in cells pretreated with CAM for 48 h but only become evident after a further 24 h of pretreatment, i.e., after a total of 72 h of pretreatment. The mechanism underlying the difference between the effects of pretreatment with CAM for 48 h and those of pretreatment with CAM for 72 h remains unclear. Although it has been reported that the influx of CAM into epithelial cells is completed within 5 min [48], in the present study the levels of intracellular CAM gradually changed over time and did not reach a plateau until after 72 h incubation of the cells with CAM (Fig. 8). The results showed that the cells incubated with 10 μM CAM had a significantly higher intracellular CAM concentration compared to the cells incubated with 5 μM CAM. Since EM has been found to exacerbate the oxidant/antioxidant balance in cells when the concentration in the extracellular fluid exceeds 6.8 μM [25], it is possible that the same changes occur in cells during pretreatment with 10 μM CAM, through an unknown mechanism. A decrease in Nrf2 activity might be involved in this mechanism.

There is no evidence to indicate whether the exposure of cells to CAM for 24, 48 or 72 h can be a model for the effect of short-term or long-term administration of CAM in the clinical setting. Furthermore, it is difficult to explain the difference between its effects on cells and its effects in clinical treatments. Nevertheless, low-dose, long-term clinical administration of CAM is believed to enhance anti-oxidative defense reactions during periods of oxidative stress. On the other hand, there is the problem that low-dose, long-term therapy of CAM would encourage the development of CAM-resistant pathogens and micoflora [49]. However, the usefulness and feasibility of CAM therapy lies in its anti-inflammatory effect rather than in its antimicrobial action [50]. Therefore, if, in addition to its anti-inflammatory effect CAM therapy also results in induction of CAM-resistant bacteria, the risk of such an increase in resistant bacteria could be reduced with the combined use of other antibacterial agents.

## Conclusions

This study showed that low-dose, long-term CAM treatment could inhibit H_2_O_2_-induced IL-8 production by suppressing transcription factor NF-κB activation in SAECs, and could inhibit H_2_O_2_-induced reduction of the GSH/GSSG ratio in SAECs via the maintenance of GSH levels through an effect on γ-GCS expression that was associated with Nrf2 mRNA expression. These data indicate that CAM is efficacious against cell dysfunction caused by oxidative stress under low-dose, long-term treatment conditions. Although it is not possible to directly translate a pharmacological effect on cells to treatment efficacy in clinical practice, the present study presents the first possible evidence of why low-dose, long-term macrolide therapy is effective against chronic inflammatory airway diseases.

## Additional files

Additional file 1: Effect of CAM treatment on the viability of SAECs. Supplementary data and statistical analysis for Figure S1a. (XLSX 32 kb)
Additional file 2: Cell viability following H_2_O_2_ treatment. Supplementary data and statistical analysis for Figure S1b. (XLSX 26 kb)
Additional file 3: Production of IL-8 by H_2_O_2_ stimulation after CAM pretreatment. Supplementary data and statistical analysis for Figure S2a. (XLSX 30 kb)
Additional file 4: Expression of IL-8 mRNA following H_2_O_2_ stimulation after CAM pretreatment. Supplementary data and statistical analysis for Figure S2b. (XLSX 31 kb)
Additional file 5: NF-κB activation induced by H_2_O_2_ after CAM pretreatment. Supplementary data and statistical analysis for Figure S3a. (XLSX 32 kb)
Additional file 6: IκBα phosphorylation induced by H_2_O_2_ after CAM pretreatment. Supplementary data and statistical analysis for Figure S3b. (XLSX 24 kb)
Additional file 7: GSH/GSSG ratio induced by H_2_O_2_ after CAM pretreatment. Supplementary data and statistical analysis for Figure S4a. (XLSX 24 kb)
Additional file 8: Expression of γ-GCS by H_2_O_2_ stimulation after CAM pretreatment. Supplementary data and statistical analysis for Figure S4b. (XLSX 25 kb)
Additional file 9: Expression of Nrf2 mRNA by H_2_O_2_ stimulation after CAM pretreatment. Supplementary data and statistical analysis for Figure S5. (XLSX 24 kb)
Additional file 10: Cell viability following H_2_O_2_ stimulation after BSO pretreatment. Supplementary data and statistical analysis for Figure S6a. (XLSX 29 kb)
Additional file 11: Cell viability following H_2_O_2_ stimulation after CAM pretreatment. Supplementary data and statistical analysis for Figure S6b. (XLSX 24 kb)
Additional file 12: Production of ROS following H_2_O_2_ stimulation after CAM pretreatment. Supplementary data and statistical analysis for Figure S7. (XLSX 24 kb)
Additional file 13: Time course of extracellular CAM concentration. Supplementary data and statistical analysis for Figure S8a. (XLSX 33 kb)
Additional file 14: Time course of intracellular CAM concentration. Supplementary data and statistical analysis for Figure S8b. (XLSX 32 kb)

## Abbreviations

AMTB: Amphotericin B
ANOVA: One-way analysis of variance
BSO: Buthionine sulfoximine
CAM: Clarithromycin
DPBS: Dulbecco’s phosphate-buffered saline
DTNB: 5,5’-dithiobis(2-nitrobenzoic acid)
ELISA: Enzyme-linked immunosorbent assay
EM: Erythromycin
GAPDH: Glyceraldehyde-3-phosphate dehydrogenase
GM: Gentamicin
GSH: Glutathione
GSSG: Oxidized glutathione
H_2_O_2_: Hydrogen peroxide
ICAM: Intracellular adhesion molecule
IL: Interleukin
IκBα: Inhibitor κBα
LPS: Lipopolysaccharide
NADPH: Reduced nicotinamide adenine dinucleotide phosphate
NF: Nuclear factor
Nrf2: Nuclear factor erythroid 2-related factor 2
PBS: Phosphate-buffered saline
PCR: Polymerase chain reaction
p-IκBα: Phosphorylated inhibitor κBα
ROS: Reactive oxygen species
SABM: Small airway basal medium
SAEC: Human small airway epithelial cell
SAGM: Small airway growth medium
TNF: Tumor necrosis factor
WST-8: 2-(2-methoxy-4-nitrophenyl)-3-(4-nitrophenyl)-5-(2,4- disulfophenyl)-2H-tetrazolium
γ-GCS: γ-glutamylcysteine synthetase
